# *TP53* mutation is associated with selective upregulation of *LAG3* independent of immune infiltration and antigen-presentation signatures in WHO grade 4 glioma

**DOI:** 10.1093/noajnl/vdag233

**Published:** 2026-09-03

**Authors:** Youji Uemae, Shunichiro Miki, Erika Yamada, Alexander Zaboronok, Tadao Ohno, Eiichi Ishikawa

**Affiliations:** Cell-Medicine, Inc., 2-1-6 Sengen, Tsukuba, Ibaraki, 305-0047, Japan (Y.U., T.O.); Department of Neurosurgery, Institute of Medicine, University of Tsukuba, 1-1-1 Tennodai, Tsukuba, Ibaraki, 305-8575, Japan (S.M., E.Y., E.I.); Department of Neurosurgery, Institute of Medicine, University of Tsukuba, 1-1-1 Tennodai, Tsukuba, Ibaraki, 305-8575, Japan (S.M., E.Y., E.I.); Department of Neurosurgery, University of Tsukuba Hospital, University of Tsukuba, 2-1-1 Amakubo, Tsukuba, Ibaraki, 305-8576, Japan (A.Z.); Cell-Medicine, Inc., 2-1-6 Sengen, Tsukuba, Ibaraki, 305-0047, Japan (Y.U., T.O.); Department of Neurosurgery, Institute of Medicine, University of Tsukuba, 1-1-1 Tennodai, Tsukuba, Ibaraki, 305-8575, Japan (S.M., E.Y., E.I.)

**Keywords:** biomarker, glioma, immune checkpoint, LAG3, TP53

## Abstract

**Background:**

Lymphocyte activation gene 3 (*LAG3*) has emerged as a clinically actionable immune checkpoint target in oncology, but its molecular determinants in glioma remain poorly characterized. We aimed to determine whether *TP53* mutation is associated with *LAG3* expression, whether this is selective among immune checkpoint genes, and whether it persists after adjustment for transcriptomic immune scores.

**Methods:**

We analyzed gene expression and mutation data from the Genomic Data Commons (GDC), incorporating three datasets (TCGA, CPTAC, HCMI) into a GDC discovery cohort of WHO grade 4 glioma (*n* = 442). An independent Glioma Longitudinal Analysis (GLASS) dataset served as a validation cohort (*n* = 79). *LAG3* and 6 additional checkpoint genes were compared between *TP53*-mutant and wild-type tumors using Wilcoxon rank-sum tests and adjusted linear regression with transcriptomic immune scores as covariates.

**Results:**

*TP53* mutation was consistently associated with elevated *LAG3* expression in the GDC cohort (median difference = +0.288 log_2_(TPM + 1), Cliff’s δ  =  0.289, *P* = 7.13 × 10^–7^) and replicated in the GLASS cohort (δ  =  0.395, *P* = .005). Among 7 checkpoint genes, only *LAG3* showed significant *TP53*-associated upregulation after Benjamini–Hochberg correction in both cohorts. The *TP53* regression coefficient was unchanged after immune score adjustment, and *TP53* status was not associated with any immune score (all *P* ≥ .67). Adjustment for tumor purity and proliferation-related Hallmark programs partially attenuated but did not abolish the association.

**Conclusions:**

*TP53* mutation is associated with selective, immune context-independent *LAG3* upregulation in grade 4 glioma, supporting *TP53* mutation as a candidate stratifier for LAG3-targeted immunotherapy and motivating mechanistic studies.

Key Points

- *TP53* mutation associates with elevated *LAG3* in grade 4 glioma across 2 cohorts.
- *LAG3* upregulation is selective among 7 checkpoint genes and context-independent.
- *TP53* mutation is a candidate biomarker for LAG3-targeted immunotherapy in glioma.

Importance of the Study

Lymphocyte activation gene 3 (LAG3)-targeted immunotherapy has recently demonstrated clinical benefit in melanoma, and multiple trials are currently evaluating LAG3 blockade in glioma. However, no molecular biomarker has been established to identify glioma patients most likely to benefit. In this study, we show across 2 independent cohorts totaling 521 grade 4 glioma cases that *TP53* mutation is associated with selective upregulation of *LAG3,* but not 6 other immune checkpoint genes, in a manner independent of bulk immune infiltration, antigen presentation signatures, tumor purity, and proliferation-related transcriptional programs. These findings identify *TP53* mutation as a candidate molecular stratifier for LAG3-targeted immunotherapy in glioma and provide a reproducible ­transcriptomic rationale for prospective biomarker evaluation in ongoing and future trials of LAG3-targeted therapies.

Glioblastoma (GBM), the most aggressive primary brain tumor in adults, presents significant therapeutic challenges despite multimodal treatment approaches. Standard therapy consists of maximal safe resection followed by concurrent temozolomide (TMZ) and radiotherapy;^1^ however, median overall survival remains approximately 15 months, with a 5-year survival rate below 10%.^1-3^ The immunosuppressive tumor microenvironment in GBM poses a major barrier to effective immunotherapy.^4^ Among immune checkpoints, lymphocyte activation gene 3 (LAG3) has emerged as a critical negative regulator of T-cell function, binding to major histocompatibility complex (MHC) class II molecules and suppressing T-cell activation and proliferation.^5-7^ LAG3 is frequently co-expressed with Programmed Cell Death protein-1 (PD-1) on tumor-infiltrating lymphocytes, and dual blockade of LAG3 and PD-1 has shown synergistic activity in preclinical models,^8^^,^^9^ and has demonstrated clinical benefit in advanced melanoma.^10^^,^^11^

Our group has developed an autologous formalin-fixed tumor vaccine (AFTV) and evaluated it in clinical trials in patients with GBM.^12-15^ Although overall efficacy was not confirmed in the full analysis set, exploratory findings suggested potential heterogeneity of benefit by molecular context, including p53 status. Specifically, in a matched cohort analysis, p53-negative status by immunohistochemistry (IHC) and gross total resection (GTR) were identified as ­predictive factors for favorable outcomes with AFTV treatment.^16^ These clinical observations prompted us to investigate the molecular basis linking *TP53* mutational status to immune checkpoint expression in GBM.

*TP53* is the most frequently mutated gene in human cancers, with mutations occurring in approximately 30% of primary GBMs.^17^^,^^18^ While immune checkpoint proteins such as PD-1, cytotoxic T-lymphocyte-associated protein 4 (CTLA-4), and LAG3 have emerged as therapeutic targets in various cancers, their relationship with *TP53* alterations remains incompletely understood. In salivary gland carcinomas, LAG3 expression on tumor-infiltrating lymphocytes was significantly associated with *TP53* mutations, particularly in *TP53* null-type tumors.^19^ However, whether this *TP53*–*LAG3* association is selective for LAG3 or extends to other checkpoint molecules, and whether it operates in glioma, has not been established. Mutant p53 proteins exhibit diverse properties beyond loss of wild-type tumor suppressor activity, including alterations in immune signaling pathways.^20-22^ Emerging evidence suggests that *TP53* mutations can modulate the tumor immune microenvironment through multiple mechanisms, including regulation of cytokine/chemokine programs and immune-evasion phenotypes;^23-25^ however, the specific relationship between *TP53* mutation status and *LAG3* expression in glioma remains unclear.

A key interpretive challenge in bulk RNA sequencing (RNA-seq)–based studies of immune checkpoint expression is the potential confounding by immune cell abundance: tumors with greater T-cell infiltration express higher levels of most checkpoint genes by cell composition alone. The antigen presentation machinery (APM), comprising MHC class I molecules (HLA-A, HLA-B, HLA-C), β2-microglobulin (B2M), and peptide transporters (TAP1, TAP2, TAPBP), is a principal determinant of the immune-engaged context in solid tumors.^26^^,^^27^ The degree of T-cell infiltration varies substantially across GBM, ranging from immune-infiltrated to immune-excluded phenotypes,^28^ and can be quantified using transcriptomic cytolytic signatures. Whether the association between *TP53* mutation and *LAG3* expression simply reflects higher immune infiltration or represents a molecularly distinct, immune-quantity–independent regulatory relationship is a critical question for understanding its biological significance and therapeutic implications.

In this study, we investigated the relationship between *TP53* mutation status and *LAG3* expression in glioma using 2 independent cohorts: a Genomic Data Commons (GDC) discovery cohort comprising WHO grade 4 glioma cases (*n* = 442) derived from 3 datasets (TCGA, CPTAC, and HCMI), and an independent Glioma Longitudinal Analysis (GLASS) cohort used for external validation (*n* = 79). We demonstrate that *TP53* mutation is associated with selectively elevated *LAG3* expression across cohorts and WHO grades, that this association is specific to *LAG3* among 7 immune checkpoint genes, and that it persists after adjustment for bulk immune infiltration and antigen presentation signatures. These findings identify a reproducible LAG3-high phenotype in *TP53*-mutant glioma that is not explained by immune cell abundance alone and support prospective evaluation of *TP53* mutation status as a candidate biomarker for LAG3-targeted immunotherapy.

## Methods

### Study Cohorts

#### GDC Discovery Cohort

Transcriptomic and somatic mutation data for glioma were obtained from the National Cancer Institute (NCI) GDC (accessed September 29, 2025), incorporating three datasets: The Cancer Genome Atlas (TCGA), the Clinical Proteomic Tumor Analysis Consortium (CPTAC), and the Human Cancer Models Initiative (HCMI). Analyses were restricted to WHO grade 2, 3, and 4 gliomas with available RNA-seq data and *TP53* mutation annotation. The primary analysis cohort comprised grade 4 tumors (*n* = 442), including TCGA (*n* = 245) and CPTAC/HCMI analyzed jointly as a non-TCGA cohort (*n* = 197). Because these datasets were generated under legacy histologic grading schemes, our primary “grade 4” analyses include a small number of isocitrate dehydrogenase (IDH)-mutant tumors. We therefore adjusted all GDC models for IDH status and additionally performed sensitivity analyses restricted to IDH-wildtype tumors (consistent with the WHO CNS5 definition of GBM, IDH-wildtype), which yielded directionally consistent results. Grade 2 (*n* = 305) and grade 3 (*n* = 204) tumors were analyzed as secondary subgroups. Gene expression values were normalized as log2(TPM + 1), where TPM denotes transcripts per million.

#### GLASS Validation Cohort

The GLASS dataset was used for independent external validation (accessed December 16, 2025).^29^ Cases were restricted to those with a GLASS sample identifier prefix, available whole-exome sequencing (WXS) data, and no overlap with TCGA (non-TCGA cases only), yielding a validation cohort of *n* = 79 (*TP53-*mutant: *n* = 24; *TP53* wild-type: *n* = 55). Expression values were normalized as log_2_(TPM + 1), consistent with the GDC cohort.

### Variable Definitions

*TP53* mutation status was coded as a binary variable (mutant = 1, wild-type = 0) in both cohorts. In the GDC cohort, the cohort was included as a binary variable indicating dataset of origin (TCGA = 1, CPTAC/HCMI = 0, with CPTAC/HCMI serving as the reference). IDH mutation status was coded as a binary variable (IDH-mutant = 1, IDH-wild-type = 0). For *TP53* mutation subtype analysis in WHO grade 4 glioma, mutations were classified into 4 groups: wild-type, hotspot missense (R175, R248, R273, R282), truncating (nonsense, frameshift, and splice-site variants), and other missense.^20^^,^^21^

### Primary Statistical Analysis

The association between *TP53* mutation status and *LAG3* expression was assessed using 2-sided Mann–Whitney U tests (Wilcoxon rank-sum tests). Effect sizes were quantified using Cliff’s delta (δ) and the Hodges–Lehmann estimator of the location shift.

#### Multiple-Testing Correction

For checkpoint specificity analyses (Section: Checkpoint gene specificity), Benjamini–Hochberg (BH) correction was applied within each cohort across the 7 checkpoint genes (*LAG3*, *PDCD1*, *CD274*, *PDCD1LG2*, *CTLA4*, *TIGIT*, *HAVCR2*). For the residual-based specificity analysis, BH correction was applied to the same 7 genes within the GDC WHO grade 4 cohort. For the *TP53* mutation subtype analysis in grade 4 glioma, overall group differences were first assessed using the Kruskal–Wallis test; BH correction was then applied to 3 prespecified pairwise comparisons versus wild-type (hotspot vs wild-type, truncating vs wild-type, other missense vs wild-type).

Linear regression was used to estimate the adjusted effect of *TP53* on *LAG3* expression. The baseline model (M0) in the GDC cohort was specified as:

*LAG3 ∼ TP53* + cohort + IDH

The baseline model in the GLASS cohort was specified as:

LAG3 ∼ TP53

IDH status was not included as a covariate in the GLASS cohort owing to the small number of IDH-mutant cases (*n* = 4). Regression coefficients (β) are reported with 95% confidence intervals (CIs) and 2-sided *P*-values.

### Meta-Analysis

To pool *TP53* regression coefficients across cohorts, fixed-effect and random-effects meta-analyses were performed using the R package *metafor* (version 4.8.0). The 3 analytic units were the TCGA cohort (GDC), the CPTAC/HCMI cohort (GDC), and the GLASS cohort (WXS). Cohort-specific regression coefficients and standard errors from models adjusted for IDH status (and cohort, where applicable) were used as input. The random-effects model was estimated using restricted maximum likelihood (REML). Between-cohort heterogeneity was quantified using I^2^.

### Immune Transcriptomic Scores

Three composite transcriptomic scores were computed as the arithmetic mean of log_2_(TPM + 1) expression values across constituent genes: a T-cell infiltration score (8 genes: *CD3D*, *CD3E*, *CD3G*, *CD8A*, *CD8B*, *GZMA*, *GZMB*, *PRF1*),^30^ an APM score (8 genes: *B2M*, *TAP1*, *TAP2*, *TAPBP*, *HLA-A*, *HLA-B*, *HLA-C*, *NLRC5*),^26^ and an interferon-gamma (IFN-γ) score (11 genes: *STAT1*, *IRF1*, *IRF9*, *CXCL9*, *CXCL10*, *CXCL11*, *GBP1*, *GBP2*, *GBP4*, *GBP5*, *IDO1*).^31^ All 27 constituent genes were present in the expression matrix, with no missing values.

To assess whether the *TP53*–*LAG3* association was explained by immune cell abundance or immune activation, sequential adjustment models were fitted:

M1: *LAG3 ∼ TP53* + cohort + IDH + T-cell score + APM score

M2: *LAG3 ∼ TP53* + cohort + IDH + T-cell score + APM score + IFN-γ score

The change in the *TP53* coefficient relative to M0 (Δβ) was computed as the primary measure of confounding by immune cell abundance. Collinearity between the T-cell and APM scores was assessed prior to model fitting (Pearson’s r = 0.511).

### Checkpoint Gene Specificity

The analysis of *LAG3* selectivity was performed across seven immune checkpoint genes: *LAG3*, *PDCD1*, *CD274*, *PDCD1LG2*, *CTLA4*, *TIGIT*, and *HAVCR2*. For each gene, both 2-sided Mann–Whitney U tests and adjusted linear regression were performed within each cohort. BH correction was applied within each cohort across the seven genes. Cohort-specific regression coefficients were derived from the primary baseline models (GDC: *LAG3* ∼ *TP53 *+ cohort + IDH; GLASS: *LAG3* ∼ *TP53* only). Progressive immune-score, tumor-purity, and proliferation-program adjustments are reported in Supplementary Figures S7 and S12 and Supplementary Table S5.

### Robustness and Sensitivity Analyses

The following analyses were performed to assess the robustness of the primary finding.

#### Immune Score Interaction and Estimated Marginal Means

To formally test the hypothesis that the *TP53* effect is consistent across levels of immune infiltration (parallel shift), interaction terms were added to the regression model: *TP53 *× T-cell score and *TP53 *× APM score. Estimated marginal mean (EMM) differences in *LAG3* expression between *TP53*-mutant and wild-type tumors were computed at representative low, mean, and high values of each immune score.

#### Immune Strata Analysis

Samples were stratified into immune-high and immune-low subgroups using mean ± 1 standard deviation (SD) cutoffs applied separately to T-cell and APM scores (T-cell: low < 0.573, high > 1.941; APM: low < 6.061, high > 7.610). The *TP53* regression coefficient for *LAG3* was estimated within each stratum.

#### Residual Approach

Each checkpoint gene was residualized against a model including T-cell score, APM score, IFN-γ score, cohort, and IDH status. Residuals were then regressed on *TP53* mutation status, and BH correction was applied across the 7 genes.

#### Cohort-Dependent Interaction

An interaction model was fitted to test whether the *TP53* effect differed between the TCGA and CPTAC/HCMI cohorts:


LAG3 ∼ TP53× cohort + IDH

This model fitted with and without immune-score adjustment (T-cell + APM). The significance of the interaction term was evaluated using both likelihood-ratio ANOVA (comparing models with and without the interaction term) and the Wald test.

#### Quantile Regression

To assess whether the *TP53* association was consistent across the distribution of *LAG3* expression, quantile regression was performed at τ  =  0.25, 0.50, and 0.75 using the R package *quantreg*. The model specification matched M0 (*TP53 *+ cohort + IDH). Bootstrap confidence intervals were computed using R = 1,000 resamples (seed = 42).

#### Permutation Test

To evaluate the stability of the *TP53*-cohort interaction under label shuffling, TP53 labels were permuted *N* = 20,000 times (seed = 42). For each permutation, the interaction t-statistic was recomputed from the same model. The permutation *P*-value was calculated as the proportion of permuted t-statistics whose absolute value exceeded that of the observed t-statistic. This procedure was applied to both the unadjusted and immune-adjusted interaction models.

#### Standardized Effect Sizes

Glass’s Δ (using the wild-type group standard deviation as the standardizer), Cohen’s *d*, and Cliff’s δ were computed for the GDC WHO grade 4 cohort overall and separately by cohort. Bootstrap 95% confidence intervals were obtained using B = 2,000 resamples (seed = 42).

#### Batch-Effect Sensitivity (ComBat)

Because the GDC discovery cohort combines samples from distinct source datasets, we assessed whether residual technical differences between sources could affect the *TP53* estimate beyond the cohort covariate. Expression data for the analyzed genes (the 27 immune-score genes and *LAG3*), together with the top 5,000 highly variable genes, were adjusted for source (TCGA vs CPTAC/HCMI) using ComBat (R package sva), protecting *TP53* and IDH status as biological covariates of interest (seed = 42). The *TP53* regression models were then refit on ComBat-adjusted *LAG3* expression, and the change in the *TP53* coefficient (Δβ%) relative to the uncorrected model was quantified.

#### Tumor Purity and Proliferation Program Adjustment

Tumor purity was estimated using the ESTIMATE algorithm. To evaluate whether tumor composition or proliferative state could account for the *TP53*–*LAG3* association, extended regression models were fitted by adding tumor purity and the top-ranked proliferation-related Molecular Signatures Database (MSigDB) Hallmark gene set scores (G2M checkpoint, MYC targets v1, E2F targets). Attenuation of the *TP53* coefficient (Δβ%) relative to the immune-adjusted base model was then quantified. As a sensitivity analysis restricted to the TCGA subset, tumor purity was additionally estimated using DNA-based ABSOLUTE calls (TCGA PanCanAtlas), matched to cases by TCGA patient barcode; for cases with multiple samples, the primary tumor and the high-confidence (“called”) ABSOLUTE solution were prioritized. ABSOLUTE purity was available for 234 of 245 TCGA grade-4 cases. Within this subset, the immune-adjusted *TP53* model for *LAG3* was refitted substituting ABSOLUTE for ESTIMATE purity, and the attenuation of the *TP53* coefficient (Δβ%) was compared between the two purity estimates. ABSOLUTE purity is not available for the CPTAC/HCMI or GLASS cohorts, which therefore retained ESTIMATE-derived purity.

### Software

All analyses were performed in R (version 4.5.1). Key packages included: *metafor* (version 4.8.0) for meta-analysis; *quantreg* for quantile regression; and *ggplot2* and *patchwork* for visualization. Figures were exported at a resolution of at least 450 dpi. All random seeds were set to 42 for reproducibility.

### Use of Artificial Intelligence

During the preparation and revision of this work, the authors used the generative AI assistant Claude (Anthropic) in a conversational manner for the following delimited purposes: (i) assistance in drafting, debugging, and documenting the R scripts used to perform the analyses and to generate the figures; (ii) assistance in drafting and language editing of the manuscript text; and (iii) assistance in preparing supporting documents, including the response to reviewers and the documentation of the public code repository.

The AI tool had no access to, and did not process any of the underlying data. All analyses were specified and executed locally by the authors in R/RStudio, and all statistical results, their verification, and their interpretation were produced by the authors. Every AI-assisted output—code and text alike—was independently reviewed, tested where applicable, and revised by the authors, who take full responsibility for the content and integrity of this work. The AI tool does not meet the criteria for authorship and is not listed as an author.

## Results

### TP53 Mutation Is Associated with Elevated LAG3 Expression in WHO Grade 4 Glioma across Independent Cohorts

We first examined the association between *TP53* mutation status and *LAG3* expression in WHO grade 4 glioma. In the GDC discovery cohort (*n* = 442), *TP53*-mutant tumors showed significantly higher *LAG3* expression than wild-type tumors (median difference = +0.288 log_2_(TPM + 1), Wilcoxon rank-sum *P* = 7.13 × 10^−7^; Cliff’s δ  =  0.289). Cohort-stratified analyses confirmed consistent direction in both TCGA (median difference = +0.338, Cliff’s δ  =  0.326, Wilcoxon rank-sum *P* = 3.78 × 10^−5^) and CPTAC/HCMI (median difference = +0.193, Cliff’s δ  =  0.234, Wilcoxon rank-sum *P* = 7.08 × 10^−3^; Figure 1A). Linear regression adjusting for cohort and IDH status confirmed the association (β = +0.262 [95% CI, +0.140, +0.383], *P* = 2.70 × 10^−5^; Supplementary Table S1). Independent validation in the GLASS cohort (WXS-only, non-TCGA cases; *n* = 79) confirmed the association (median difference = +0.573, *P* = 5.49 × 10^−3^; δ  =  0.395, Figure 1B). Meta-analysis pooling the 3 datasets (GDC-TCGA, GDC-CPTAC/HCMI, and GLASS) showed a consistent positive effect (random-effects β = +0.308 [95% CI, +0.098, +0.518], *P* = .004; I^2^ = 69.0%, Figure 1C). Similar directionality was observed across WHO grades 2 and 3 (grade 2: median difference = +0.215, p_BH = 1.72 × 10^−4^, δ  =  0.261; grade 3: median difference = +0.189, p_BH = 7.92 × 10^−3^, δ  =  0.217; Supplementary Figure S1), consistent with a grade-independent trend. In these secondary analyses, *P*-values were adjusted for multiple testing across the 2 prespecified grade comparisons (grades 2 and 3) using the BH procedure.

**Figure 1.**
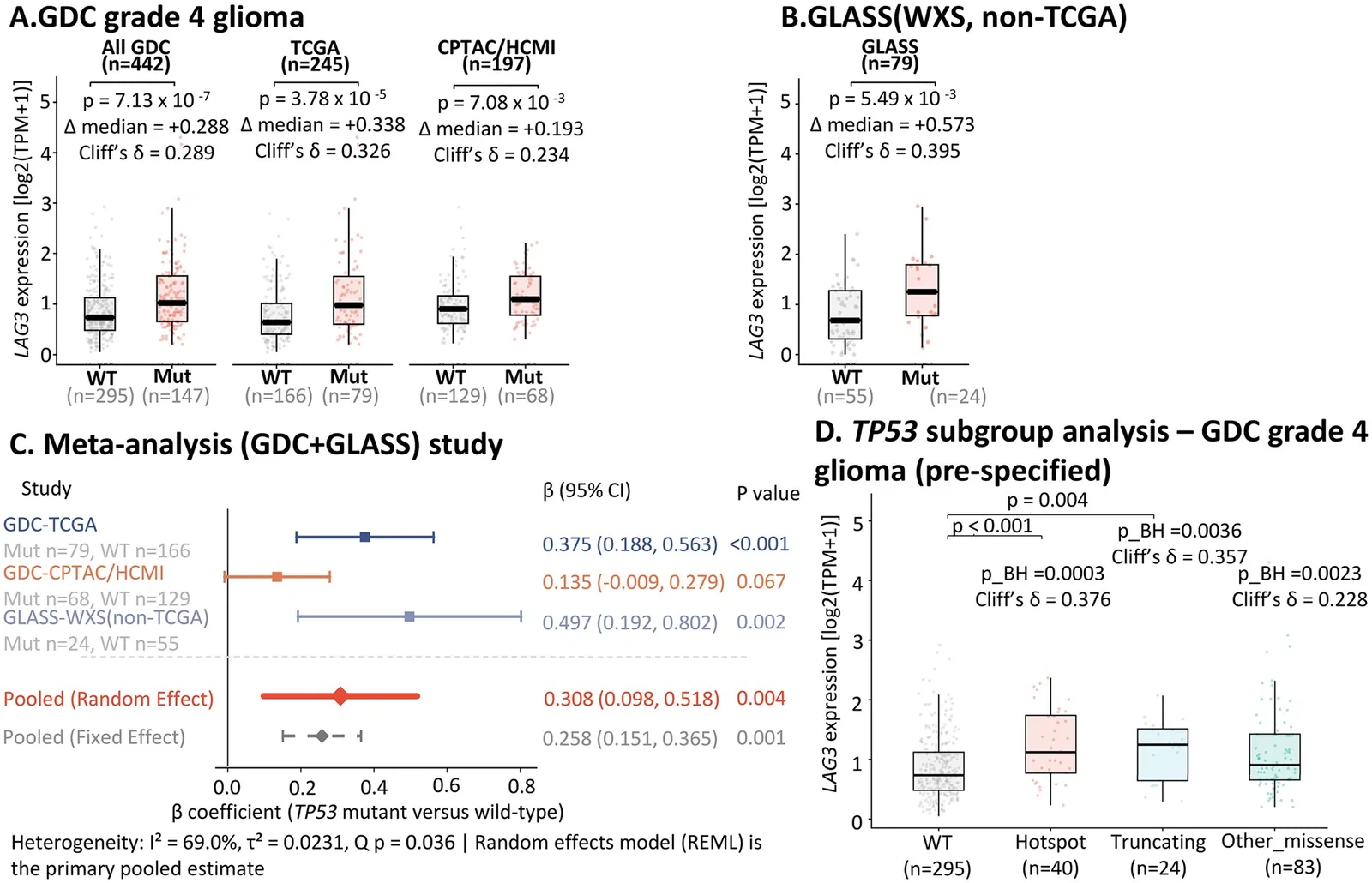
*TP53* mutation is associated with increased *LAG3* expression in WHO grade 4 glioma across independent cohorts. (A) *LAG3* expression by *TP53* mutation status in GDC WHO grade 4 glioma, shown for the combined cohort (All GDC; *n* = 442; *TP53*-mutant: *n* = 147, wild-type: *n* = 295) and stratified by cohort (TCGA: *n* = 245; CPTAC/HCMI: *n* = 197). Boxes indicate median and interquartile range; whiskers extend to 1.5× IQR. *P*-values are from 2-sided Wilcoxon rank-sum tests (unadjusted); effect sizes are Cliff’s δ (All GDC: δ = 0.289; TCGA: δ = 0.326; CPTAC/HCMI: δ = 0.234). (B) *LAG3* expression by *TP53* status in the independent GLASS validation cohort (WXS-only, non-TCGA cases; *n* = 79). Plot conventions as in panel A. (C) Forest plot of *TP53* regression coefficients (β) for *LAG3* across three cohorts (GDC-TCGA, GDC-CPTAC/HCMI, GLASS), with fixed-effect and random-effects pooled estimates. Cohort-specific regression coefficients are derived from the primary baseline models (GDC-TCGA and GDC-CPTAC/HCMI: LAG3 ∼ TP53 + IDH, fitted separately within each cohort; GLASS: *LAG3* ∼ *TP53* only). Progressive immune-score, tumor-purity, and proliferation-program adjustments are reported in Supplementary Figures S7 and S12 and Supplementary Table S5. Points indicate β; error bars indicate 95% confidence intervals. Confidence intervals in this forest plot are computed by a normal (Wald) approximation (z = 1.96), consistent with the meta-analysis framework, whereas the intervals for the same coefficients in the regression tables (Supplementary Table S1; Supplementary Figure S7) use the t-distribution; point estimates and *P*-values are identical between the two (eg for the GLASS cohort, β = +0.497 corresponds to [+0.192, +0.802] by normal approximation and [+0.187, +0.807] by the t-distribution). (D) *LAG3* expression by *TP53* mutation class in GDC WHO grade 4 glioma (wild-type, hotspot, truncating, other missense). Prespecified pairwise comparisons versus wild-type are annotated with Benjamini–Hochberg (BH)-adjusted *P*-values calculated across the 3 prespecified mutant-versus-wild-type comparisons (hotspot, truncating, and other missense vs wild-type); Cliff’s δ values are shown for each comparison.

As a secondary analysis, all 3 *TP53* mutation classes—hotspot, truncating, and other missense—showed elevated *LAG3* compared with wild-type (Cliff’s δ = 0.376, 0.357, and 0.228, respectively; all p_BH < 0.004; BH correction across the three mutant-vs-wild-type comparisons; Figure 1D), suggesting the association is not restricted to a single mutation class. The positive *TP53* effect was observed consistently across quantiles of the *LAG3* distribution and was not attributable to outliers (Supplementary Figure S2).

### TP53-Associated Upregulation Is Selective for LAG3 among Immune Checkpoint Genes

To assess whether the *TP53* association reflected a broader immune checkpoint program, we evaluated seven checkpoint genes (*LAG3*, *PDCD1*, *CD274*, *PDCD1LG2*, *CTLA4*, *TIGIT*, *HAVCR2*) in both cohorts (Figure 2). In GDC, WHO grade 4 cohort (*n* = 442), after BH correction within the cohort across the 7 checkpoint genes, only *LAG3* showed a significant *TP53*-associated increase by both Wilcoxon test (p_BH = 4.99 × 10^−6^) and adjusted regression (β = +0.262 [95% CI, +0.140, +0.383], p_BH = 1.89 × 10^−4^); all other checkpoint genes were not significant (all regression p_BH > 0.71). For completeness, log_2_ fold-changes (as the difference in median log_2_(TPM + 1) expression, *TP53*-mutant vs wild-type) for all 7 checkpoint genes in both cohorts are reported in Supplementary Table S2. *LAG3* showed the largest positive change (log_2_ fold-change based on group medians = +0.288 in the GDC grade-4 cohort and +0.573 in GLASS), whereas the remaining 6 genes ranged from −0.196 to +0.075 in the GDC cohort. In the GLASS cohort (n = 79), *LAG3* again remained significant after BH correction (Wilcoxon p_BH = 0.038; regression β = +0.497 [95% CI, +0.187, +0.807], p_BH = 0.014), whereas no other gene showed consistent *TP53*-associated upregulation. *PDCD1* showed a cohort-specific nominal decrease in CPTAC/HCMI by Wilcoxon testing, but this was not consistent across cohorts and was not supported by covariate-adjusted regression, reinforcing the reproducible selectivity for *LAG3*. This selectivity was further supported by an orthogonal residual approach in which immune-score variation was removed prior to testing (Supplementary Figure S3; Supplementary Table S2).

**Figure 2.**
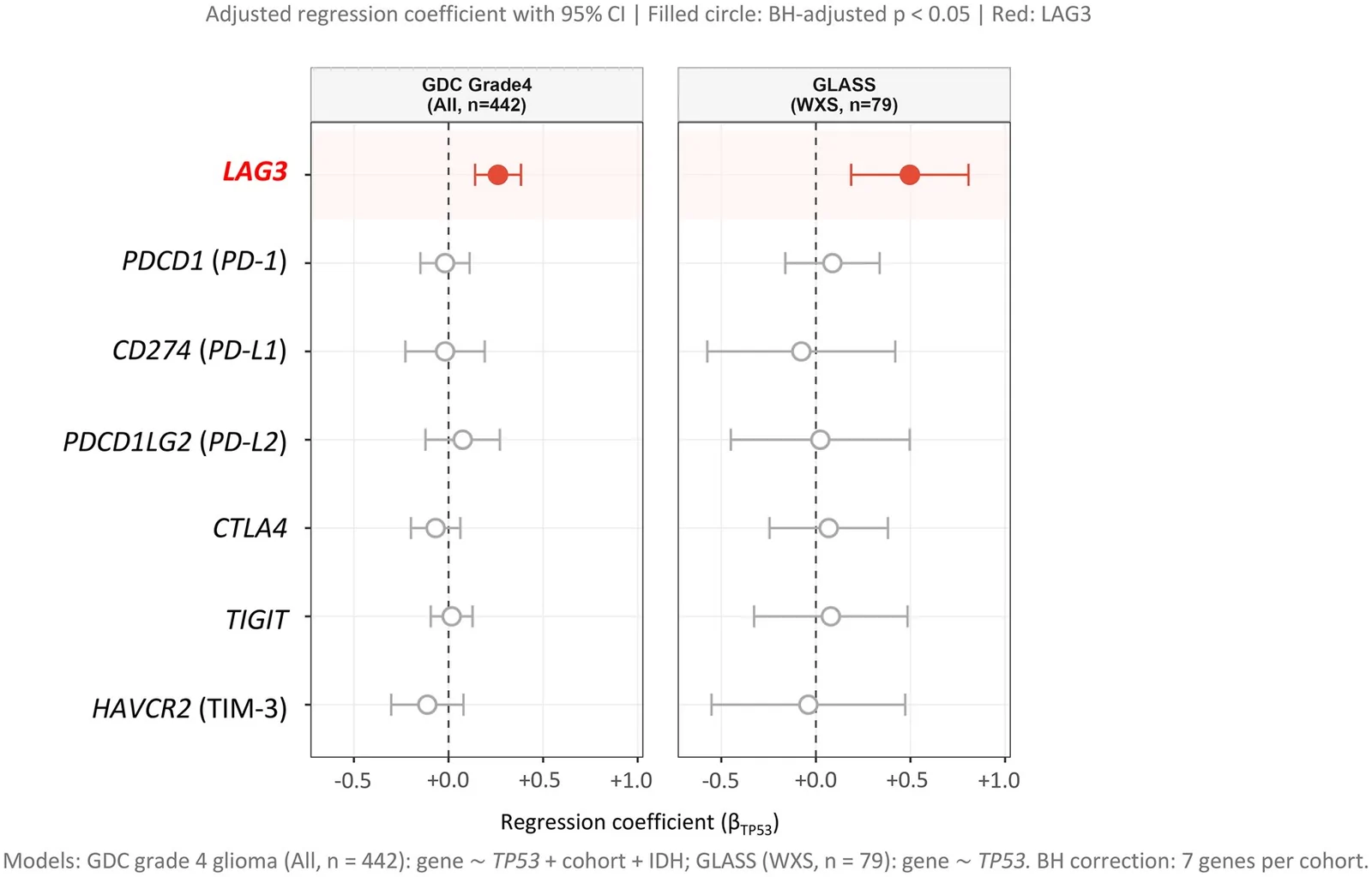
*TP53* mutation selectively associates with *LAG3* among seven immune checkpoint genes. Adjusted regression coefficients (β_*TP53*_) with 95% confidence intervals for the association of *TP53* mutation with the expression of seven immune checkpoint genes. Results are shown for GDC WHO grade 4 glioma (*n* = 442; adjusted for cohort and IDH status) and the GLASS validation cohort (WXS) (*n* = 79; modeled with *TP53* status as the sole predictor). Benjamini–Hochberg (BH) correction was applied within each cohort across the seven genes. Filled symbols indicate BH-adjusted *P* < .05. Genes are ordered from top to bottom: *LAG3*, *PDCD1*, *CD274*, *PDCD1LG2*, *CTLA4*, *TIGIT*, *HAVCR2*. Complete statistics are provided in Supplementary Table S2; residual-based confirmation is shown in Supplementary Figure S3.

### The TP53–LAG3 Association Is Not Explained by Immune Infiltration or Antigen Presentation Signatures

Because bulk RNA-seq reflects varying immune cell content, we tested whether the *TP53*–*LAG3* association could be accounted for by immune cell abundance or activation. We computed 3 transcriptomic scores from log_2_(TPM + 1) values: a T-cell infiltration score (mean of 8 genes, including *CD3D*, *CD8A*, *GZMA*), an APM score (mean of 8 genes, including *B2M*, *TAP1*, *HLA-A*), and an IFN-γ score (mean of 11 genes, including *STAT1*, *CXCL9*, *IDO1*). Each score showed a moderate positive correlation with *LAG3* (vs T-cell: r = 0.406; vs. APM: r = 0.374; vs IFN-γ: r = 0.381; Figure 3A), and together substantially increased the explained variance in *LAG3* (R^2^ from 0.080 to 0.313), confirming that immune context is a major determinant of bulk *LAG3* expression. T-cell and APM scores were also moderately correlated (r = 0.511), consistent with a partially shared immune activation structure.

**Figure 3.**
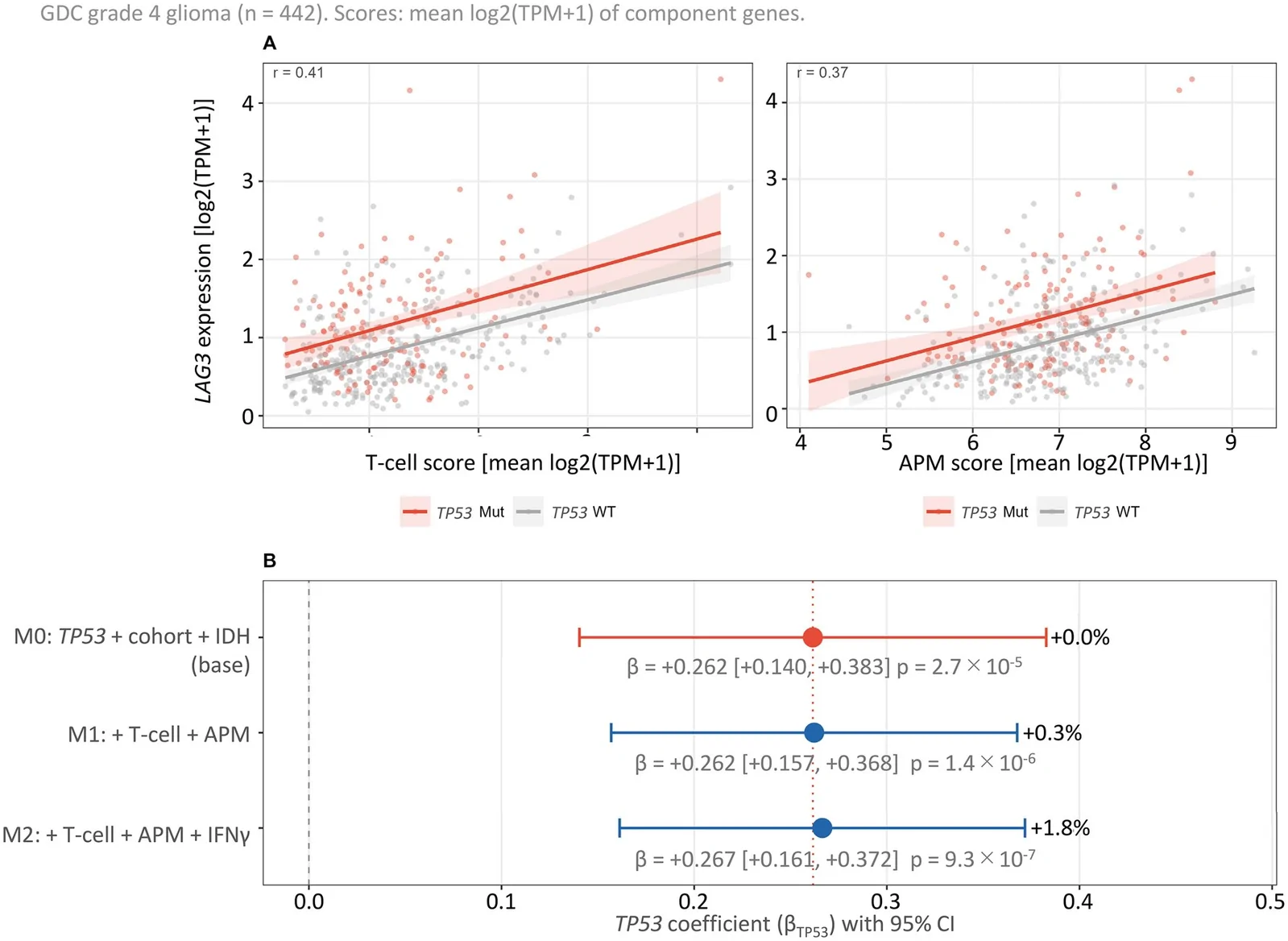
The *TP53*–*LAG3* association persists after adjustment for immune infiltration and antigen presentation signatures. (A) Scatter plots of *LAG3* expression versus T-cell infiltration score (left) and antigen presentation machinery (APM) score (right) in GDC WHO grade 4 glioma (*n* = 442), stratified by *TP53* mutation status. Immune scores were computed as the arithmetic mean of log_2_(TPM + 1) values for constituent genes (T-cell score: 8 genes; APM score: 8 genes). Lines indicate linear fits for each group. Pearson correlation coefficients with *LAG3* are annotated (T-cell: r = 0.406; APM: r = 0.374). The IFN-γ score is incorporated in panel B (model M2) but not shown as a scatter plot. (B) Stability of the *TP53* regression coefficient across successive immune-score adjustment models in GDC WHO grade 4 glioma. GDC models: M0 = *TP53* + cohort + IDH status; M1 = M0 + T-cell score + APM score; M2 = M1 + IFN-γ score (11 genes). Complete M0 regression coefficients for all terms are provided in Supplementary Table S1, Part 1. Points indicate β; error bars indicate 95% confidence intervals. Percentage values indicate the change in the *TP53* coefficient relative to M0. The corresponding immune-adjustment sequence for the GLASS validation cohort (*n* = 79), in which M0 = *LAG3* ∼ *TP53* only, is shown in Supplementary Figure S7.

However, adjusting for these scores did not attenuate the *TP53* coefficient. The *TP53* estimate was essentially unchanged from the baseline model M0 (β = +0.262) to M1 incorporating T-cell and APM scores (β = +0.262, *P* = 1.4 × 10^−6^; change = +0.3%) and to M2 additionally including the IFN-γ score (β = +0.267, *P* = 9.3 × 10^−7^; change = +1.8%; Figure 3B; Supplementary Table S1). This stability was consistent with the near-identical immune score distributions observed between *TP53*-mutant and wild-type tumors (T-cell score median: 1.128 vs 1.130; APM: 6.841 vs 6.874; IFN-γ: 3.087 vs 3.153; Supplementary Figure S4; Supplementary Table S3), and *TP53* status was not associated with any immune score in adjusted regression (all *P*  ≥ .67). The association remained positive across all 4 immune strata and significant in 3 of 4 (APM high: p_BH = 0.170), with no significant TP53-immune score interaction (Supplementary Figures S5 and S6). In the independent GLASS cohort, the *TP53* coefficient similarly remained positive and significant after immune-score adjustment, with only modest attenuation (M1: β = +0.459, change = −7.6%; M2: β = +0.465, change = −6.3%; both *P* < .002; Supplementary Figure S7).

The *TP53* effect size was larger in TCGA than in CPTAC/HCMI (regression β = +0.375 vs +0.135; Glass’s Δ  =  0.768 vs 0.363; Supplementary Figures S8 and S11), a pattern attributable in part to broader *LAG3* dispersion in TCGA (wild-type SD = 0.543 vs 0.486 in CPTAC/HCMI; Supplementary Figure S8). Formal interaction testing suggested a possible cohort-dependent difference in effect magnitude that reached ­statistical significance after immune-score adjustment (unadjusted: interaction β = −0.223, *P* = .059; immune-adjusted: β = −0.247, *P* = .017; permutation *P* = .015; Supplementary Figures S9 and Figure S10; Supplementary Table S4). However, both cohorts showed directionally consistent positive *TP53* estimates (TCGA: β = +0.365 [95% CI, +0.204, +0.527]; CPTAC/HCMI: β = +0.142 [95% CI, −0.031, +0.315]; Supplementary Figure S9), and because this comparison involves only 2 cohorts, we interpret this as supportive sensitivity evidence rather than a definitive heterogeneity claim. Further adjustment for ESTIMATE-derived tumor purity modestly attenuated the *TP53* coefficient in GLASS (β: 0.465 → 0.427; Δβ = −8.3%), consistent with the GDC finding (Δβ = −9.7%). Additional adjustment for proliferation-related Hallmark programs (G2M checkpoint, MYC targets v1, E2F targets) produced further but comparable attenuation in both cohorts, each expressed relative to its own immune-adjusted base model (GDC: maximum Δβ = −26.0%, β  =  0.197, *P* = 1.4 × 10^−4^; GLASS: maximum Δβ = −28.3%, β  =  0.334, *P* = .021); in both cohorts the *TP53*–*LAG3* ­association remained positive and statistically significant (Supplementary Table S5), supporting the robustness of the finding to tumor composition adjustment in an independent cohort (Supplementary Figure S12; Supplementary Table S5). Explicit batch correction across source datasets did not materially change the association: after ComBat adjustment protecting *TP53* and IDH, the *TP53* coefficient for *LAG3* was essentially unchanged in both the baseline model (β = +0.262 → +0.243; Δβ = −7.0%; *P* = 9.3 × 10^−5^) and the immune-adjusted model (β = +0.262 → +0.242; Δβ = −7.9%; *P* = 1.3 × 10^−5^; Supplementary Table S7), indicating that the cohort covariate already accounted for between-source differences.

## Discussion

*TP53* mutation is among the most frequent somatic alterations in glioma, yet its relationship to the immune microenvironment remains incompletely characterized. In the present study, we demonstrate that *TP53* mutation is consistently associated with elevated *LAG3* expression across independent glioma cohorts, that this association is selective for *LAG3* among 7 immune checkpoint genes, and that it persists after adjustment for bulk immune infiltration and antigen presentation signatures. Together, these findings identify a reproducible *LAG3*-high phenotype in *TP53*-mutant grade 4 glioma that is not explained by immune cell abundance alone.

The reproducibility of the *TP53*–*LAG3* association across the GDC cohort (*n* = 442) and the independent GLASS cohort (*n* = 79), as well as across WHO grades 2, 3, and 4, argues against a cohort-specific or grade-restricted phenomenon. Meta-analysis confirmed a consistent positive effect across three cohorts (random-effects β = +0.308, *P* = .004), despite moderate between-cohort heterogeneity (I^2^ = 69%), which was at least partially attributable to cohort-specific differences in *LAG3* dispersion rather than directional inconsistency. The observation that all 3 *TP53* mutation subtypes (hotspot, truncating, and other missense) showed elevated LAG3 compared with wild-type further suggests that the association is not restricted to gain-of-function hotspot mutations but may reflect a broader consequence of *TP53* loss of function. This is consistent with established roles of *TP53* in transcriptional repression and chromatin remodeling,^20^ which could influence the expression of immune regulatory genes through mechanisms independent of specific mutation topology.

The selectivity of the *TP53* association for *LAG3* among 7 immune checkpoint genes is a central and unexpected finding. In both the GDC and GLASS cohorts, *LAG3* was the only gene to show a consistent and statistically significant *TP53*-associated increase after BH correction, whereas *PDCD1*, *CD274*, *CTLA4*, *TIGIT*, *HAVCR2*, and *PDCD1LG2* showed no consistent upregulation. This selectivity was confirmed through an orthogonal residual approach: after removing variation attributable to T-cell, APM, and IFN-γ scores, only *LAG3* residuals retained a significant *TP53* association. LAG3 differs mechanistically from PD-1 and CTLA-4 in several respects, including its primary ligands (MHC class II, FGL1, galectin-3), its role in regulatory T-cell function, and its reported responsiveness to transcription factors associated with T-cell exhaustion, including TOX and nuclear factor of activated T cells (NFAT), which are downstream of sustained antigen stimulation.^32^ Whether *TP53* mutation influences *LAG3* through direct transcriptional regulation, via altered chromatin accessibility at the *LAG3* locus, or indirectly through p53-regulated downstream effectors remains to be determined and constitutes a tractable hypothesis for future mechanistic investigation.

A key concern in bulk RNA-seq analyses of immune checkpoint expression is confounding by immune cell abundance: tumors with greater lymphocyte infiltration will exhibit higher expression of checkpoint genes simply due to differences in cellular composition. Our data addresses this concern in several complementary ways. First, the *TP53* regression coefficient for *LAG3* was essentially unchanged after adjustment for T-cell, APM, and IFN-γ scores (β change: +0.3% to +1.8%), despite these scores substantially increasing the explained variance (R^2^ from 0.080 to 0.313). Second, *TP53*-mutant and wild-type tumors showed nearly identical immune score distributions, and *TP53* status was not associated with any immune score in adjusted regression (all *P* ≥ .67), excluding the possibility that *TP53* mutation simply marks immunologically hotter tumors. Third, stratified analyses across immune-high and immune-low subgroups confirmed that the *TP53* effect on *LAG3* was significant in three of 4 strata, with EMM differences that were consistent rather than stratum-dependent. Taken together, these analyses suggest that the elevated *LAG3* in *TP53*-mutant tumors reflects a qualitative difference in immune checkpoint expression, a shift in the regulatory state of LAG3, rather than a quantitative difference in immune cell infiltration. Consistent with these findings, additional adjustment for tumor purity and proliferation-related transcriptional programs partially attenuated, but did not abolish, the *TP53*–*LAG3* association in both cohorts. To confirm that this purity adjustment did not depend on the expression-based ESTIMATE metric, we repeated it in the TCGA subset (*n* = 234) using an orthogonal, DNA-based purity estimate (ABSOLUTE); the 2 estimates were positively correlated (Pearson r = 0.73), and substituting ABSOLUTE for ESTIMATE produced an essentially identical, modest attenuation of the *TP53* coefficient (Δβ = −12.4% vs −13.1% for ESTIMATE), with *TP53* remaining positive and highly significant (β = +0.337, 95% CI, +0.186 to +0.487; *P* = 1.6 × 10^−5^; Supplementary Table S6).

The cohort-dependent attenuation of effect size warrants careful interpretation. The *TP53* coefficient was larger in TCGA (β = +0.375) than in CPTAC/HCMI (β = +0.135), and this difference reached statistical significance in the immune-adjusted interaction model (*P* = .017; permutation *P* = .015). However, both cohorts showed directionally consistent positive estimates, and the attenuation in CPTAC/HCMI was accompanied by narrower dispersion of *LAG3* expression (wild-type SD: 0.49 vs 0.54 in TCGA) and smaller standardized effect sizes. This pattern is consistent with technical differences in library preparation, RNA extraction, or sequencing depth between platforms, which can compress expression variance and attenuate effect size estimates without reversing direction. The GLASS cohort, derived from an independent sequencing platform, showed the largest point estimate (median difference = +0.573; δ  =  0.395), further supporting the consistent direction of the association. We therefore interpret the cohort interaction as a precision and scale difference rather than biological heterogeneity, while acknowledging that the comparison of only two GDC cohorts limits definitive conclusions.

Several limitations of this study merit consideration. First, all analyses are based on bulk RNA-seq, which reflects the aggregate transcriptional output of a mixed cellular population. The cellular source of elevated LAG3, whether tumor-infiltrating T cells, regulatory T cells, natural killer cells, or tumor cells themselves, cannot be determined from bulk data alone. Single-cell RNA-seq and spatial transcriptomics approaches will be necessary to resolve which cell populations drive the *TP53*-associated *LAG3* signal and whether this reflects increased *LAG3* expression per cell or increased abundance of LAG3-high cell types. In particular, the cellular compartment expressing LAG3—for example, exhausted or regulatory T cells versus tumor cells—cannot be assigned from bulk data, and single-cell or spatial profiling stratified by *TP53* status is the appropriate next step to resolve this. Such an analysis requires single-cell or single-nucleus datasets with matched, patient-level *TP53* mutation calls (eg from whole-exome or whole-genome sequencing); to our knowledge, adequately powered public resources meeting this requirement were not available at the time of this study. We also sought to corroborate the transcriptional signal at the protein level using the CPTAC GBM global proteome (tandem mass tag (TMT) mass spectrometry; 10,997 proteins across 99 tumors), but *LAG3* ­protein was not detected in any case, including under its aliases LAG-3/CD223. This reflects the detection limit of bulk mass spectrometry rather than a quantitative failure: canonical high-abundance controls (EGFR, GFAP, VIM, B2M, GAPDH, ACTB) were detected in all 99 tumors, whereas among the 7 checkpoint proteins only the more abundant CD274 (99/99) and HAVCR2 (90/99) were detectable, and lymphocyte-restricted markers were detected only spor­adically (Supplementary Table S8). Low-abundance, lymphocyte-restricted proteins such as LAG3 therefore fall below the sensitivity of bulk proteomics, which underscores the value of the transcriptomic approach for detecting this regulation and the need for targeted protein-level validation. Second, the cross-sectional nature of these cohorts precludes causal inference; *TP53* mutation status and *LAG3* expression were measured at a single time point, and the direction of the regulatory relationship remains to be established experimentally. Third, the GLASS cohort used for validation was restricted to grade 4 WXS-only non-TCGA cases (*n* = 79), limiting statistical power for subgroup analyses within that cohort. Fourth, although we observe consistent associations across grade 2, 3, and 4 tumors, the grade-stratified analyses were not the primary focus of this study, and formal grade-by-*TP53* interaction testing was not performed. Fifth, the GDC cohorts were annotated under legacy histologic grading schemes predating the WHO CNS5 integrated diagnostic framework; complete reclassification incorporating *TERT* mutation, *EGFR* amplification, chromosome +7/−10, and *CDKN2A*/*B* status was not feasible given the available data. The adjustment for IDH status in all primary models and the IDH-wild-type sensitivity analyses partially mitigate this limitation, but the results should be interpreted in the context of histologic rather than fully integrated molecular grading. Functional and protein-level validation in patient tissue—for example, IHC for LAG3 stratified by *TP53* status—will be required to establish whether the elevated transcript translates into increased protein and to localize its cellular source; such validation is a priority for ongoing and future work.

Despite these limitations, the present findings have potential translational relevance. LAG3 has emerged as a clinically actionable immune checkpoint target; relatlimab (anti-LAG3) plus nivolumab has received regulatory approval in melanoma, and multiple LAG3-directed agents are under active clinical investigation in neuro-oncology, including an ongoing randomized phase II trial of relatlimab plus nivolumab versus lomustine in recurrent GBM (ClinicalTrials.gov: NCT06325683). In a murine GBM model, Harris-Bookman et al. reported that *LAG3* expression emerges early during T-cell exhaustion and that initiating LAG3 blockade earlier, including in combination with anti-PD-1, was more effective than later initiation, consistent with LAG3 marking an early stage of T-cell exhaustion.^33^ This preclinical observation suggests that, beyond target selection, the timing of LAG3-directed intervention relative to the evolving exhaustion state may influence therapeutic activity. In this context, *TP53* mutation status, associated here with a reproducible *LAG3*-high phenotype, may help define a molecular subgroup in which LAG3-based strategies warrant focused evaluation, while acknowledging that prospective validation in treated cohorts is required.

In summary, we present multi-cohort evidence that *TP53* mutation is associated with selective upregulation of *LAG3* in grade 4 glioma, independent of bulk immune infiltration. This association is reproducible, checkpoint-specific, and robust across both quantitative and nonparametric analytical frameworks. These findings motivate both mechanistic dissection of the *TP53*–*LAG3* regulatory relationship and prospective evaluation of *TP53* mutation status as a potential biomarker for LAG3-targeted immunotherapy in glioma.

## Supplementary Material

vdag233_Supplementary_Data

## Data Availability

The datasets analyzed in this study are publicly available: TCGA/GDC grade-4 glioma RNA-seq and mutation data from the NCI Genomic Data Commons (https://portal.gdc.cancer.gov); CPTAC glioblastoma proteomic data via cBioPortal (dataset gbm_cptac_2021, https://www.cbioportal.org); GLASS data from the GLASS consortium (Synapse: syn17038081); and ABSOLUTE tumor-purity calls from the TCGA PanCanAtlas (https://gdc.cancer.gov/about-data/publications/pancanatlas). All analysis code used to generate the results and figures is openly available at https://github.com/y-uemae/NOA-D-26-00229R1 and archived at Zenodo (https://doi.org/10.5281/zenodo.21257112).
