# Supplementary material for: TP53 mutation is associated with selective upregulation of LAG3 independent of immune infiltration and antigen-presentation signatures in WHO grade 4 glioma
Source: Neurooncol Adv. 2026 Sep 3;8(1):vdag233. doi: 10.1093/noajnl/vdag233 (PMC13629437; doi:10.1093/noajnl/vdag233)
Supplement: Supplementary file 1 [file vdag233_supplementary_data.docx]

**TP53 mutation is associated with selective upregulation of LAG3 independent of immune infiltration and antigen-presentation signatures in WHO grade 4 glioma**

**Supplementary Figure S1. *TP53* mutation is associated with elevated *LAG3* expression across WHO grades 2 and 3 glioma.**

*LAG3* expression by *TP53* mutation status in GDC WHO grade 2 (n = 305; *TP53*-mutant: n = 144, wild-type: n = 161) and grade 3 (n = 204; *TP53*-mutant: n = 91, wild-type: n = 113) glioma. Boxes indicate median and interquartile range; whiskers extend to 1.5× IQR. P values are Benjamini–Hochberg (BH)-adjusted from two-sided Wilcoxon rank-sum tests (grade 2: median difference = +0.215, p_BH = 1.72 × 10⁻⁴, Cliff’s δ = 0.261; grade 3: median difference = +0.189, p_BH = 7.92 × 10⁻³, Cliff’s δ = 0.217), consistent with the grade 4 primary finding.

**Supplementary Figure S2. Quantile regression confirms the *TP53* effect across the *LAG3* distribution**

Quantile regression coefficients for *TP53* at τ = 0.25, 0.50, and 0.75, with bootstrap 95% confidence intervals (R = 1,000; seed = 42), alongside the OLS estimate for reference. All quantile estimates are positive and statistically significant (τ = 0.25: β = +0.163 [95% CI: +0.053, +0.273], p = 0.004; τ = 0.50: β = +0.203 [95% CI: +0.066, +0.340], p = 0.004; τ = 0.75: β = +0.405 [95% CI: +0.195, +0.615], p = 0.0002), indicating that the *TP53*–*LAG3* association is not driven by outliers or confined to a specific part of the distribution.

**Supplementary Figure S3. Residual analysis confirms *LAG3* specificity after removing immune-score variation**

Each of the seven checkpoint genes was residualized against T-cell score, APM score, IFN-γ score, cohort, and IDH status. Residuals were then regressed on *TP53* mutation status. Only *LAG3* residuals showed a significant positive *TP53* association after BH correction across the seven genes (β = +0.242 [95% CI: +0.142, +0.341], p_BH < 0.0001). All other checkpoint genes were not significant (all p_BH > 0.48). Genes are ordered as in Figure 2. Points indicate β; error bars indicate 95% confidence intervals.

**Supplementary Figure S4. Immune scores do not differ by *TP53* status**

(A) T-cell infiltration score, APM score, and IFN-γ score by *TP53* mutation status in GDC WHO grade 4 glioma (n = 442; *TP53*-mutant: n = 147; *TP53* wild-type: n = 295), shown as violin plots with overlaid boxplots. Medians were nearly identical between groups (T-cell: 1.128 vs. 1.130; APM: 6.841 vs. 6.874; IFN-γ: 3.087 vs. 3.153).

(B) Regression coefficients for *TP53* predicting each immune score, adjusted for cohort and IDH status, with 95% confidence intervals. No immune score showed a significant association with *TP53* status (T-cell: β = −0.009 [95% CI: −0.151, +0.133], p = 0.904; APM: β = +0.010 [95% CI: −0.152, +0.171], p = 0.908; IFN-γ: β = −0.039 [95% CI: −0.218, +0.140], p = 0.670), indicating that *TP53* mutation does not materially shift bulk immune infiltration or activation as measured by these transcriptomic scores.

**Supplementary Figure S5. Estimated marginal means confirm a parallel shift of the *TP53* effect across immune-score levels**

Estimated marginal mean (EMM) differences in *LAG3* expression (*TP53*-mutant minus wild-type) at representative low, mean, and high values of the T-cell score (left panel) and APM score (right panel), with all other covariates held at their reference values. Neither *TP53* × T-cell (β_int = +0.053, p = 0.505) nor *TP53* × APM interaction term (β_int = +0.033, p = 0.627) was statistically significant, supporting an approximately parallel shift of *TP53*-mutant versus wild-type *LAG3* expression across immune-score levels. Points indicate EMM differences; error bars indicate 95% confidence intervals.

**Supplementary Figure S6. The *TP53*–*LAG3* association is robust across immune strata**

*TP53* regression coefficients for *LAG3* within immune subgroups defined by T-cell score (low/high) and APM score (low/high), using mean ± 1 SD as cutoffs (T-cell: low < 0.573, high > 1.941; APM: low < 6.061, high > 7.610). The overall coefficient from the unstratified model is shown as a reference line. The *TP53* effect on *LAG3* remains positive and statistically significant in three of four strata (T-cell low: β = +0.509, p_BH = 0.021; T-cell high: β = +0.581, p_BH = 0.029; APM low: β = +0.454, p_BH = 0.004). The APM high stratum showed a positive but not significant association (β = +0.545, p_BH = 0.170). Points indicate β; error bars indicate 95% confidence intervals. p_BH, BH-adjusted p value (28 tests).

**Supplementary Figure S7. Immune-score adjustment in the GLASS validation cohort**

Stability of the *TP53* regression coefficient in the GLASS WXS-only non-TCGA cohort (n = 79) across successive immune-score adjustment models. M0: *TP53* status only (β = +0.497 [95% CI: +0.187, +0.807], p = 0.002). M1: M0 + T-cell score + APM score (β = +0.459 [95% CI: +0.184, +0.733], Δβ= −7.6%, p = 0.001). M2: M1 + IFN-γ score (β = +0.465 [95% CI: +0.189, +0.742], Δβ= −6.3%, p = 0.001). The GLASS model did not include IDH status as a covariate owing to the small number of IDH-mutant cases (n = 4). Panel layout mirrors Figure 3B.

**Supplementary Figure S8. Cohort differences in *LAG3* distribution and estimation precision**

**(A)** *LAG3* expression distributions by *TP53* status within TCGA (n = 245) and CPTAC/HCMI (n = 197), shown as density plots. *LAG3* exhibited broader dispersion in TCGA than in CPTAC/HCMI (wild-type SD: 0.543 vs. 0.486; mutant SD: 0.840 vs. 0.476).

**(B)** *TP53* regression coefficients with 95% confidence intervals by cohort (TCGA: β = +0.375; CPTAC/HCMI: β = +0.135). The *TP53* effect is directionally consistent across both cohorts; the larger confidence interval in TCGA is consistent with its greater dispersion of *LAG3* expression.

**Supplementary Figure S9. *TP53*-by-cohort interaction analysis and cohort-specific estimates**

**(A)** Interaction model results testing whether the *TP53* effect on *LAG3* differs between TCGA and CPTAC/HCMI (model: *LAG3* ~ *TP53* × cohort + IDH). The interaction term (*TP53* × cohort) was borderline without immune-score adjustment (β = −0.223 [95% CI: −0.455, +0.009], p = 0.059; ANOVA F = 3.58, df = 1) and reached statistical significance after adjustment for T-cell and APM scores (β = −0.247 [95% CI: −0.448, −0.045], p = 0.017). Points indicate β; error bars indicate 95% confidence intervals.

**(B)** Cohort-specific *TP53* effects estimated from the interaction model (TCGA: β = +0.365 [95% CI: +0.204, +0.527]; CPTAC/HCMI: β = +0.142 [95% CI: −0.031, +0.315]), indicating effect attenuation rather than reversal in CPTAC/HCMI. Given that this comparison involves only two cohorts within the GDC dataset, results are reported as sensitivity evidence.

**Supplementary Figure S10. Permutation test for the *TP53*-by-cohort interaction**

Null distributions of the interaction t-statistic from N = 20,000 permutations of *TP53* labels (seed = 42), shown for models without immune-score adjustment (A) and with immune-score adjustment (B; covariates: T-cell score, APM score). Observed t-statistics are indicated by dashed vertical lines (unadjusted: t_obs = −1.893, 94.9th percentile; immune-adjusted: t_obs = −2.403, 98.5th percentile). Permutation p values are 0.051 (unadjusted) and 0.015 (immune-adjusted), consistent with the parametric results in Supplementary Figure S9. The permutation null distributions were approximately normal (mean ≈ 0, SD ≈ 0.97), supporting the assumption of label exchangeability.

**Supplementary Figure S11. Standardized effect sizes are positive across metrics and cohorts**

Glass's Δ, Cohen's *d*, and Cliff's δ for the *TP53*–*LAG3* association in GDC WHO grade 4 glioma, shown for the combined cohort and separately by cohort, with bootstrap 95% confidence intervals (B = 2,000 resamples; seed = 42). In the combined cohort, Glass's Δ = 0.590 [95% CI: 0.333, 0.875], Cohen's *d* = 0.529 [95% CI: 0.334, 0.745], Cliff's δ = 0.289. TCGA showed larger standardized effects (Glass's Δ = 0.768 [95% CI: 0.397, 1.191]) than CPTAC/HCMI (Glass's Δ = 0.363 [95% CI: 0.080, 0.732]); all estimates were positive with confidence intervals not crossing zero in the combined cohort and TCGA, and directionally consistent in CPTAC/HCMI.

**Supplementary Figure S12. Adjustment for tumor purity and proliferation-related programs** **attenuates but does not abolish the *TP53*–*LAG3* association in both GDC and GLASS cohorts**

**(A)** *TP53* regression coefficient (β) for *LAG3* across four extended adjustment models in the GDC discovery cohort (n = 442; blue) and the GLASS validation cohort (n = 79; red). Extended models (E0–E3) were fitted on top of the immune-adjusted base model: E0 included *TP53* status, T-cell score, APM score, and IFN-γ score (plus cohort and IDH status in GDC); E1 added ESTIMATE-derived tumor purity; E2 added the G2M checkpoint score; E3 added all three top-ranked proliferation scores simultaneously (G2M checkpoint, MYC targets v1, E2F targets). Points indicate β; error bars indicate 95% confidence intervals (GLASS only). Significance indicators reflect unadjusted two-sided p values for the *TP53* coefficient (*** p < 0.001, ** p < 0.01, * p < 0.05). Note that E0 here corresponds to the GDC model M2 described in Figure 3B and Supplementary Fig. S7.

**(B)** Percentage attenuation of the *TP53* coefficient (Δβ%) relative to E0 for each additional covariate set, shown separately for GDC (blue) and GLASS (red). Tumor purity adjustment produced similar attenuation in both cohorts (GDC: −9.7%; GLASS: −8.3%). Further adjustment for proliferation-related programs produced comparable attenuation in both cohorts (GDC: maximum Δβ = −26.0%; GLASS: −28.3%), yet the *TP53* coefficient remained positive and statistically significant in both cohorts at maximum adjustment (GDC: β = 0.197, p = 1.4 × 10^-4^; GLASS: β = 0.334, p = 0.021).

Immune scores were computed as the arithmetic mean of log2(TPM+1) values across constituent genes (T-cell: 8 genes; APM: 8 genes; IFN-γ: 11 genes). Proliferation scores were derived from ssGSEA on MSigDB Hallmark gene sets. Δβ% was calculated relative to E0 within each cohort.

**Supplementary Table S1. Complete M0 regression coefficients and stability of the *TP53* coefficient across successive immune-score adjustment models in GDC WHO grade 4 glioma and the GLASS validation cohort**

Part 1 shows all regression coefficients from the baseline model (M0) in GDC WHO grade 4 glioma (n = 442). Part 2 shows the stability of the *TP53* coefficient across M0, M1, and M2 in both cohorts. M1 and M2 add immune transcriptomic scores to M0; only the *TP53* coefficient is shown for M1 and M2. The GLASS M0 model includes *TP53* status only.

Cohort, coded as TCGA = 1, CPTAC/HCMI = 0 (reference); a negative coefficient indicates lower adjusted *LAG3* in TCGA relative to CPTAC/HCMI after accounting for *TP53* and IDH status; this reflects baseline expression differences rather than a reversal of the *TP53* effect, which remains positive in both cohorts within the GDC dataset (Supplementary Fig. S9B). IDH, coded as mutant = 1, wild-type = 0. The upper confidence limit for cohort [<0.001] reflects a boundary estimate at 3 decimal places; the exact value is −0.000491. β_TP53 [95% CI], regression coefficient for *TP53* mutation status with 95% confidence interval. R^2^, model coefficient of determination. Δβ vs M0, percentage change in the *TP53* coefficient relative to model M0, calculated as 100 × (β_Mx − β_M0) / |β_M0|. GDC M0 model: *LAG3* ~ *TP53* + cohort + IDH. GDC M1 model: *LAG3* ~ *TP53* + cohort + IDH + T-cell score + APM score. GDC M2 model: *LAG3* ~ *TP53* + cohort + IDH + T-cell score + APM score + IFN-γ score. GLASS M0: *LAG3* ~ *TP53* only. Immune scores were computed as the arithmetic mean of log_2_(TPM+1) values for constituent genes: T-cell score (*CD3D*, *CD3E*, *CD3G*, *CD8A*, *CD8B*, *GZMA*, *GZMB*, *PRF1*; 8 genes); APM score (*B2M*, *TAP1*, *TAP2*, *TAPBP*, *HLA-A*, *HLA-B*, *HLA-C*, *NLRC5*; 8 genes); IFN-γ score (*STAT1*, *IRF1*, *IRF9*, *CXCL9*, *CXCL10*, *CXCL11*, *GBP1*, *GBP2*, *GBP4*, *GBP5*, *IDO1*; 11 genes). APM, antigen presentation machinery; IDH, isocitrate dehydrogenase.

**Supplementary Table S2. Checkpoint gene specificity: association of *TP53* mutation with expression of seven immune checkpoint genes in GDC WHO grade 4 glioma and the GLASS validation cohort**

Abbreviations: mut, TP53-mutant; WT, TP53 wild-type; CI, confidence interval; BH, Benjamini–Hochberg; Cliff's δ, nonparametric effect size (range −1 to +1); Wilcoxon p_BH, BH-adjusted p value from a two-sided Wilcoxon rank-sum test; Reg. p_BH, BH-adjusted p value from linear regression.

ᵃ Median diff / log2FC: difference in median log₂(TPM+1) expression (TP53-mutant − wild-type). Because expression is analyzed on the log₂ scale, this difference is the log₂ fold-change between the two groups. Values are not converted to linear fold-changes, because the +1 pseudocount means that an exponentiated ratio would refer to (TPM + 1) rather than to TPM.

ᵇ β_TP53 [95% CI]: regression coefficient for TP53 mutation status (mutant = 1, wild-type = 0), with 95% confidence interval. GDC models: gene expression ~ TP53 + cohort + IDH status. GLASS model: gene expression ~ TP53 only (IDH status not included owing to the small number of IDH-mutant cases; n = 4).

ᶜ Sig.: significance indicator. *, p_BH < 0.05; ***, p_BH < 0.001; blank, not significant.BH correction was applied within each cohort across the seven genes. LAG3 entries are shown in bold.

**Supplementary Table S3. Association of *TP53* mutation status with immune transcriptomic scores in GDC WHO grade 4 glioma**

Results of linear regression models testing whether T-cell infiltration, antigen presentation machinery (APM), or IFN-γ scores differ by *TP53* mutation status. n = 442 (TP53 mutant: n = 147; *TP53* wild-type: n = 295).

Median values are shown for log_2_(TPM+1)-averaged immune scores. β_*TP53* [95% CI], regression coefficient for *TP53* mutation status (mutant = 1, wild-type = 0) with 95% confidence interval; models adjusted for cohort and IDH status. P value, from adjusted linear regression. Sig., ns (not significant, p ≥ 0.05). Immune score gene compositions are as described in Supplementary Table S1. mut, *TP53*-mutant; WT, *TP53* wild-type.

**Supplementary Table S4. Summary of sensitivity and robustness analyses: cohort-dependent interaction, permutation testing, quantile regression, and standardized effect sizes**

Summary of four complementary analyses evaluating the robustness and generalizability of the *TP53*–*LAG3* association in GDC WHO grade 4 glioma (n = 442). Results correspond to Supplementary Figures S2 and S8–S11.

For the *TP53*-by-cohort interaction analyses, the model tested whether the *TP53* effect differed between TCGA (n = 245) and CPTAC/HCMI (n = 197); the interaction term is *TP53* × cohort. The immune-adjusted interaction model additionally included T-cell and APM scores as covariates. For permutation testing, *TP53* labels were permuted N = 20,000 times (seed = 42); p_perm denotes the proportion of permuted t-statistics with absolute value exceeding the observed t-statistic. For quantile regression, bootstrap confidence intervals used R = 1,000 resamples (seed = 42); the model was *LAG3* ~ *TP53* + cohort + IDH. For standardized effect sizes, bootstrap confidence intervals used B = 2,000 resamples (seed = 42); Glass's Δ uses the wild-type group SD as the standardizer. Given that the cohort interaction analysis involves only two cohorts within the GDC dataset, all cohort-related results (interaction, permutation, standardized effects) are reported as sensitivity evidence rather than definitive heterogeneity claims. β_int, interaction term coefficient; t_obs, observed t-statistic for the interaction term; p_perm, permutation p value; τ, quantile; CI, confidence interval.

**Supplementary Table S5. Effect of tumor purity and proliferation-related Hallmark program adjustment on the *TP53* regression coefficient for *LAG3* in GDC and GLASS cohorts**

Base model(E0) — GDC: *LAG3* ~ *TP53* + T-cell score + APM score + IFN-γ score + cohort + IDH status (n = 442); GLASS: *LAG3* ~ *TP53* + T-cell score + APM score + IFN-γ score (n = 79; cohort and IDH excluded: single cohort and n = 4 IDH-mutant cases, respectively). Tumor purity was estimated using the ESTIMATE algorithm. G2M, MYC targets v1, and E2F targets scores were computed using ssGSEA on MSigDB Hallmark gene sets. Δβ%, percentage change in the *TP53* coefficient relative to the base model(E0), calculated as 100 × (β_Ex − β_E0) / |β_E0|; "—" indicates the reference model. The top-3 proliferation scores were entered simultaneously in E3; owing to their intercorrelation, coefficients in E3 should be interpreted as joint sensitivity evidence rather than independent effects. β, regression coefficient for *TP53* mutation status (mutant vs. wild-type); p, two-sided p value from linear regression; adjusted R^2^, adjusted coefficient of determination.

**Supplementary Table S6. Tumor-purity adjustment is robust to the purity-estimation method (TCGA grade-4 subset, n = 234).**

ABSOLUTE purity from the TCGA PanCanAtlas (TCGA_mastercalls.abs_tables_JSedit.fixed.txt); available for 234 of 245 TCGA grade-4 cases. ESTIMATE vs ABSOLUTE purity: Pearson r = 0.73, Spearman ρ = 0.75. All three models were fitted on the same 234 samples; Δβ is expressed relative to the immune-adjusted base model. β, linear-regression coefficient for TP53 (mutant vs wild-type) on LAG3 log2(TPM+1) expression.

**Supplementary Table S7. The *TP53*–*LAG3* association is robust to explicit cross-source batch correction (ComBat).**

ComBat batch correction (R package sva; seed = 42) with batch defined by source dataset (TCGA, n = 245; CPTAC/HCMI, n = 197), protecting TP53 and IDH status as biological covariates of interest. Correction was applied to the analyzed genes (the 27 immune-score genes and LAG3) together with the top 5,000 highly variable genes; the TP53 models were then refit on ComBat-adjusted LAG3 expression. Δβ is expressed relative to the uncorrected model.

**Supplementary Table S8. Detection of immune-checkpoint, control, and lymphocyte-restricted proteins in the CPTAC glioblastoma global proteome (n = 99 tumors).**

cBioPortal dataset gbm_cptac_2021; global proteome, TMT mass spectrometry (10,997 proteins across 99 tumors). Detection was defined as a non-missing quantification value in the global proteome matrix. Proteins reported as 0/99 were absent from the matrix entirely; HAVCR2 was present but missing in 9 tumors. An alias-aware search was applied to all seven checkpoint proteins (for LAG3: LAG-3, CD223; for PDCD1: PD-1, CD279; for PDCD1LG2: PD-L2, CD273; for CTLA4: CD152; for TIGIT: VSTM3, VSIG9; for CD274: PD-L1, B7-H1; for HAVCR2: TIM-3, CD366), and none of the five undetected proteins was present under any alias. Identical detection counts were obtained from the z-scored quantification matrix. High-abundance positive controls were detected in all tumors, whereas among the seven checkpoint proteins only the more abundant CD274 and HAVCR2 were detectable, and lymphocyte-restricted markers were detected only sporadically — consistent with an abundance-dependent detection limit of bulk mass spectrometry.
